# A synchronized multimodal neuroimaging dataset for studying brain language processing

**DOI:** 10.1038/s41597-022-01708-5

**Published:** 2022-09-30

**Authors:** Shaonan Wang, Xiaohan Zhang, Jiajun Zhang, Chengqing Zong

**Affiliations:** 1 National Laboratory of Pattern Recognition, Institute of Automation, CAS, Beijing, China; 2 School of Artificial Intelligence, University of Chinese Academy of Sciences, Beijing, China

**Keywords:** Neural encoding, Neural decoding, Language

## Abstract

We present a synchronized multimodal neuroimaging dataset for studying brain language processing (SMN4Lang) that contains functional magnetic resonance imaging (fMRI) and magnetoencephalography (MEG) data on the same 12 healthy volunteers while the volunteers listened to 6 hours of naturalistic stories, as well as high-resolution structural (T1, T2), diffusion MRI and resting-state fMRI data for each participant. We also provide rich linguistic annotations for the stimuli, including word frequencies, syntactic tree structures, time-aligned characters and words, and various types of word and character embeddings. Quality assessment indicators verify that this is a high-quality neuroimaging dataset. Such synchronized data is separately collected by the same group of participants first listening to story materials in fMRI and then in MEG which are well suited to studying the dynamic processing of language comprehension, such as the time and location of different linguistic features encoded in the brain. In addition, this dataset, comprising a large vocabulary from stories with various topics, can serve as a brain benchmark to evaluate and improve computational language models.

## Abstract (summary)

**Table:** 

Measurement(s)	functional brain measurement • Magnetoencephalography
Technology Type(s)	Functional Magnetic Resonance Imaging • Magnetoencephalography
Factor Type(s)	naturalistic stimuli listening
Sample Characteristic - Organism	humanbeings

## Background & Summary

Scientists have spent decades studying human language processing. Evidence has accumulated that the language-related cortex includes Broca’s area in the inferior frontal gyrus (IFG) and Wernicke’s area in the superior temporal gyrus (STG) as well as parts of the middle temporal gyrus (MTG) and the inferior parietal and angular gyrus (AG) in the parietal lobe^1^. Moreover, different language components maybe processed at different times^2^. However, language is a complex and dynamic system that makes it difficult to decompose natural language with traditional tightly controlled experimental designs. Moreover, the fragmented conclusions drawn by breaking apart language components are believed to be difficult to integrate to form a whole picture of human language processing.

In addition, there is a growing consensus that analyzing neuroimaging data collected by naturalistic stimuli may provide another perspective on human language processing. Compared to traditional factorial designs with highly controlled features, natural language as a stimulus is better suited to everyday use and involves dynamic changes, making it more suitable for studying dynamic language processing in the brain. This approach may provide an opportunity to study the human brain as a whole. The key to this approach is large-scale, high-quality neuroimaging data collected using natural language stimuli.

Fortunately, we are beginning to see a proliferation of public neuroimaging datasets acquired using rich, naturalistic experimental paradigms, among which most collected brain imaging data use audiovisual movie stimuli^3–6^. Few datasets are language oriented, which is optimal for studying brain language processing^7,8^. However, these studies only collected functional magnetic resonance imaging (fMRI) data, which is not sufficient for language studies because language comprehension is immediate and rapid. Multimodal neuroimaging data with higher spatiotemporal resolution are needed to examine the intricate and subject-dependent patterns of brain structural connectivity, functional connectivity, and physiological organization. Two previous works collected both fMRI and electroencephalogram (EEG)^9^ or magnetoencephalography (MEG)^10^ data. However, the two-modality data in the former study^9^ were collected from different participants, which makes it impossible to study both the time and location of language processing for a single participant. Moreover, the stimuli in the latter study^10^ included designed normal or scrambled sentences instead of natural language stories.

The dataset^11^ proposed here aims to address some of the issues mentioned above. This dataset contains neuroimaging data with both high time-resolution MEG and high space-resolution fMRI measurements, which were collected for a total of 12 participants listening to 6 hours of stories. An additional question-answering test after each story ensured that the participants listened carefully and understood the meaning of the text during recording. Both the raw and preprocessed fMRI and MEG results are provided, where the preprocessed fMRI data include both surface-based and volume-based results. To facilitate further language comprehension research, the syntactic tree structures of all story texts are annotated by human experts, and word and sentence boundaries and part-of-speech (POS) tags are provided. The audio and transcripts are automatically aligned temporally. We also provide character and word embeddings from various computational models. Furthermore, additional neuroimaging T1, T2, resting, and diffusion MRI data for each participant were acquired and included, which could represent a data foundation for studying fine-grained structural and functional language in individual brains.

Such high-quality neuroimaging data with rich annotations are very useful for studying a variety of research questions involving brain language processing, including semantic representation, syntactic processing, and attention and memory mechanisms when comprehending language. Moreover, given that the dataset contains a variety of topics and a broad vocabulary, it can also be seen as a human benchmark for evaluating computational models. For instance, these data could be used to assess whether the attention and memory variables calculated by computational models are similar to those of humans. Furthermore, this dataset could be used to improve a computational model, either by adding the data into the model training process or by building a new model architecture inspired by the findings of the relation between the model and the human brain.

## Methods

### Participants

All 12 participants were recruited from universities in Beijing; they had also engaged in our previous experiments and exhibited habits that yielded good quality data, such as having only slight head movement in the scanner, concentrating on the experiments, and having high accuracy on the questionnaires after scanning. The participants completed both fMRI and MEG visits (first fMRI and then the MEG experiments, with a gap of at least 1 month). The study participants included 4 females and 8 males, with an age range of 23–30 years (see the details in Table 1). All participants were right-handed adults with Mandarin Chinese as their native language who reported having normal hearing and no history of neurological disorders. Participants were paid and provided written informed consent. The study was conducted under the approval of the Institutional Review Board of Peking University.

**Table 1:** Details of the participants, including ID, age, sex (M: male, F: female), and behavior results (fMRI-acc: the quiz accuracy in the fMRI experiments, MEG-acc: the quiz accuracy in the MEG experiments).

Participant ID	01	02	03	04	05	06	07	08	09	10	11	12
Age	26	30	26	28	26	25	26	23	24	25	23	24
Sex	M	F	M	M	M	M	F	M	F	M	F	M
fMRI-acc (%)	94.17	89.17	92.50	82.50	85.00	89.17	93.33	94.17	95.00	95.83	89.17	94.17
MEG-acc (%)	95.00	90.00	92.50	87.50	90.00	87.5	95.00	90.83	96.67	93.33	88.33	95.00

### Experimental procedures

**Fig. 1:**
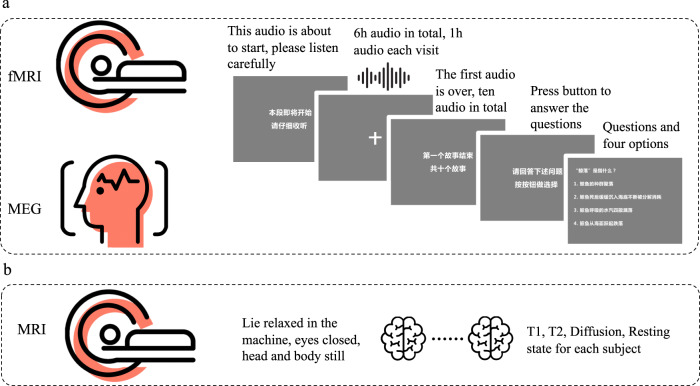
Schematic overview of the study procedure. (**a**) The participants followed the instructions on the screen and listened to stories while their brain activity was recorded by fMRI and MEG. (**b**) Participants lied in the MRI machine while structural and resting-state MRI data were recorded.

Before each scanning, participants first completed a simple informational survey form and an informed consent form. During both fMRI and MEG scanning, participants were instructed to listen and pay attention to the story (stimulus), remain still, and answer questions on the screen after the completion of each audio component. Stimulus presentation was implemented using Psychtoolbox-3. Specifically, at the beginning of each run, the instruction “Waiting for scanning” appeared on the screen, followed by a blank screen for 8 seconds. Then, as shown in Fig. 1a, the following instructions were provided: “The audio is about to start, please listen carefully”. This information appeared and lasted for 2.65 seconds before the audio started. As the audio played, a centrally located fixation marker was presented on the screen. Finally, two questions about the story were presented, each with four answers to choose from, and the time for answering was controlled by the participants. The Auditory story stimuli were delivered via S14 insert earphones for fMRI studies (with headphones or foam padding placed over the earphones to reduce scanner noise), whereas Elekta matching insert earphones were used for the MEG studies.

At each visit, a volume check was performed to ensure that participants could comfortably hear the auditory stimulus prior to data collection. The MRI recording was split into 7 visits, each lasting 1.5 hours, in which T1, T2, and resting MRI data were collected at the first visit, fMRI data obtained during listening were collected at visits 1 to 6, and diffusion MRI data were collected at the last visit. As shown in Fig. 1b, during MRI scanning, including T1, T2, diffusion and resting stages, participants were instructed to lie relaxed and still in the machine. The MEG recording was split into 6 visits, each lasting 1.5 hours. The stimuli materials are presented in the same order between participants but in a different order for MEG and fMRI visits.

### Stimuli

**Fig. 2:**
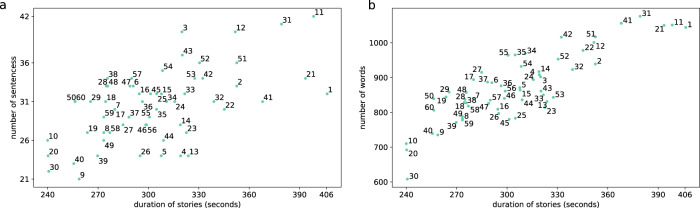
Details of the 60 stories. (**a**) The duration and number of sentences in each story. The number in the picture denotes the story number. (**b**) The duration and number of words of each story (duplicate words were also counted).

Stimuli included the audio of 60 stories that are 4 to 7 minutes long and comprise various topics, such as education and culture. All audio was downloaded from the Renmin Daily Review website and was read by the same male broadcaster at https://www.ximalaya.com/toutiao/30917322/. All stimuli were normalized using Adobe Audition software and played at 16 kHz. The corresponding text was also downloaded from the Renmin Daily Review website, and errors were manually corrected to ensure that the audio and text were aligned. As shown in Fig. 2, the durations of the 60 stories range from 240 to 406 seconds. In general, there are 52,269 words in all the stories, and each story includes 608 to 1,076 words, forming a vocabulary of 9,153 words.

### Data acquisition

#### Structural T1 and T2

All MRI data were collected at the Center for MRI Research, Peking University using a Siemens Prisma 3 T scanner with a 64-channel receiver coil. High-resolution T1-weighted MRI data were acquired using a 3D MPRAGE sequence with 0.8-mm isotropic resolution (TR = 2.4 s, TI = 1 s, TE = 2.22 ms, flip angle = 8°, 224 axial slices with thickness = 0.8 mm, axial FOV = 256 × 256 mm, and data matrix = 320 × 320). T2-weighted MRI data were acquired using the Siemens SPACE sequence^12^ with 0.8-mm isotropic resolution with the same matrix, FOV, and number of slices as used for the T1w images (TR = 3.2 s, and TE = 563 ms).

#### Diffusion MRI

The diffusion MRI images were acquired using a 2D spin-echo single-shot multiband echo-planar imaging (EPI) sequence with a multiband factor of 3 and a monopolar gradient pulse (TR = 3500 ms, TE = 86 ms, flip angle = 90°, FOV = 210 × 210 mm, 100 axial slices with thickness = 1.5 mm, and acquisition matrix = 140 × 140). A full session included 2 runs with 2 different gradient tables (as in the Human Connectome Project) collected in opposite (anterior-to-posterior and posterior-to-anterior) phase-encoding polarities with 98 and 99 diffusion-weighted directions respectively.

#### Functional and Resting MRI

BOLD-sensitive functional and resting state images were obtained using a multi-shot T2-weighted GE-EPI sequence (TR = 710 ms, TE = 30 ms, matrix size = 106 × 106, flip angle = 54°, FOV = 212 × 212 mm^2^, 72 2-mm-thick axial slices, and 634 volumes). The resting data included 2 runs with 2 different gradient tables collected in opposite (anterior-to-posterior and posterior-to-anterior) phase-encoding polarities.

#### MEG

The continuous MEG signal was recorded using a 306-channel, whole-head MEG system (Elekta-Neuromag TRIUX, Helsinki, Finland) at Peking University. Before each block started, the head position of each participant was determined by four head-position indicator (HPI) coils. Two electrooculogram (EOG) electrodes were applied to monitor the vertical and horizontal eye movements and blinks, which were attached inferior to the left eye and superior to the right eye. During recording, the MEG data were acquired with a sampling frequency of 1,000 Hz and filtered online between 0.1 Hz and 330 Hz.

### Preprocessing

#### MRI

For the MRI data, we used minimal preprocessing pipelines (HCP) at https://www.humanconnectome.org/software/hcp-mr-pipelines to preprocess the structural, functional, resting and diffusion images. This is performed on a local server cluster with native installation version scripts on Centos 7.8 operating system including 20 computing nodes (17 CPU nodes and 3 GPU nodes). Specifically, preprocessing was performed separately for each stimulus with the following steps: (1) HCP PreFreeSurfer, FreeSurfer and PostFreeSurfer pipelines were successively conducted on the T1 and T2 images. This process included correcting the gradient distortion, aligning repeated runs, removing the skull from the image, removing readout distortion, performing bias field correction, registering the image to the standard Montreal Neurological Institute space, and producing tissue maps and surface files for pia and white matter for each participant, followed by down-sampling and registering surface files. (2) After the structural preprocessing was complete, HCP fMRIVolume and fMRISurface pipelines were used on the functional and resting images. This process included removing spatial distortions, realigning volumes to compensate for participant motion, registering the fMRI data to the structural information, reducing the bias field, normalizing the 4D image to a global mean, masking the data, and transferring the time series from the volume into the CIFTI standard space. (3) HCP diffusion preprocessing pipelines also depend on the outputs generated by structural preprocessing, and the HCP pipelines performed the following steps: normalizing the b0 image intensity across runs; removing EPI distortions, eddy-current-induced distortions, and participant motion; correcting for gradient nonlinearities; registering the diffusion data with the structure; bringing the data into the 1.25 mm structural space; and masking the data with the final brain mask^13^.

Overall, this pipeline can generate high-quality cortical surfaces, segmentations and myelin maps; generate precise within- and cross-participant registration; and provide data in CIFTI format.

#### MEG

**Fig. 3:**
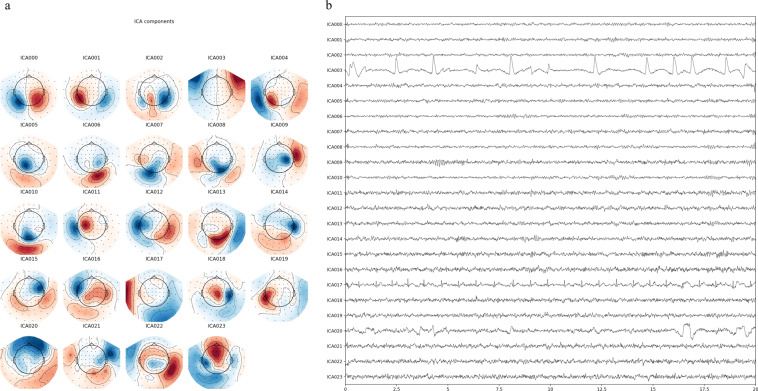
ICA component examples of the first participant on (**a**) the interpolated sensor topography and (**b**) the interpolated sensor space.

The raw data were first preprocessed off-line using the temporal signal space separation (tSSS) method implemented in Maxfilter software (Elekta-Neuromag) for magnetic artifact suppression. Then, the bad channels automatically identified by Maxfilter (3–11 channels per participant) with default threshold value were excluded by the interpolating method. The independent component analysis (ICA) method was applied to remove ocular artifacts using MNE software at http://www.martinos.org/mne/. This is performed on a local server with native installation version scripts on Ubuntu 16.04.7 LTS operating system including 2 CPU computing nodes. Specifically, as shown in Fig. 3, the ICA components with the highest correlation to the EOG (blinks, ICA003), EKG (heart beats, ICA017) and saccade (eye movement, ICA020) signals were selected and rejected. Then, a bandpass filter (0.1 to 40 Hz) was applied to the denoised data. The delay between the event timing and the actual timing of the auditory presentation to the participant was a stable 39.5 ms detected with a sound sensor.

### Annotations

**Fig. 4:**
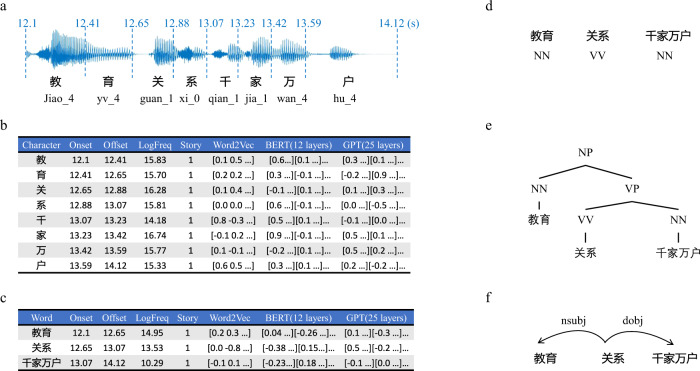
An example of annotation information for the stimuli. (**a**) Speech-to-text alignment. (**b**) Linguistic annotations of characters. (**c**) Linguistic annotations of words. (**d**) Part-of-speech tag annotations. (**e**) Constituency tree annotations. (**f**) Dependency tree annotations.

As shown in Fig. 4, in addition to the neuroimaging datasets, we provide rich annotations of the audio and text, including the following:

- **Speech-to-text alignment** (Fig. 4a) The character and word boundaries in the audio were identified and aligned to the transcripts using a pretrained GMM-HMM model^14^ in the Kaldi tools, where the model was trained with AISHELL-2 data and open-source scripts at https://github.com/kaldi-asr/kaldi/tree/master/egs/aishell2/s5. This process provided the onset and offset time of each character and word in the audio. The onset and offset time were increased by 10.65 seconds to align with the time of the fMRI images because the fMRI scan started 10.65 seconds before playing the audio. The corresponding pinyin and tone of each Chinese character were also generated using Kaldi tools.
- **Frequency** (Fig. 4b,c) The character and word frequencies were calculated from the Xinhua news corpus at http://www.xinhuanet.com/whxw.htm and then log-transformed.
- **Textual embeddings** (Fig. 4b,c) The static embeddings that are not sensitive to context were calculated by the Word2Vec model from https://code.google.com/archive/p/word2vec/. Specifically, the character-level and word-level models were trained separately on the same Xinhua news corpus (19.7 GB of text) with the same model parameters (i.e., CBOW architecture, 10 training iterations, window size of 10, embedding dimensions of 100 and 300). The dynamic character and word embeddings that are sensitive to context were calculated by using the BERT and GPT2 models. We included the 768-dimensional embeddings from all 12 layers of BERT from https://huggingface.co/bert-base-chinese and the 1024-dimensional embeddings from all 25 layers of GPT2 (the Chinese GPT2 model was trained using the corpus (250 GB of text) from https://github.com/CLUEbenchmark/CLUE and the model from https://github.com/NVIDIA/Megatron-LM).
- **Part of speech** (Fig. 4d) The POS tag of each word was annotated by experts following the criteria of PKU Chinese Treebank.
- **Syntactic tree** (Fig. 4e,f) The constituent tree structure was manually annotated by linguistic students following the PKU Chinese Treebank criteria with TreeEditor tools, and all results were double checked by different experts. Specifically, the texts were first split into sentences, and the sentences were segmented into words. Then, the part-of-speech tag of each word in the sentence was identified, and finally, the tree structures were annotated. The dependency tree structure was transformed from the constituent tree using Stanford CoreNLP tools at https://nlp.stanford.edu/software/stanford-dependencies.html, and no error was generated because the two tree structures had a one-to-one correspondence.

## Data Records

**Fig. 5:**
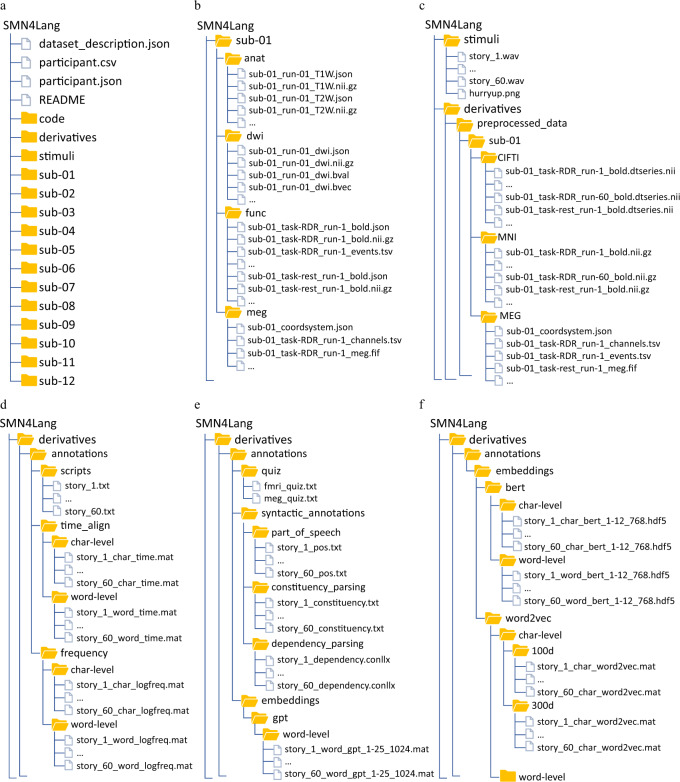
Organization of the data collection. (**a**) General overview of the directory structure. (**b**) Content of participant-specific directories. (**c**) Content of stimuli and participant-specific preprocessed data directories. (**d**) Content of the annotations directory, including scripts, aligned word and character times, and frequencies of words and characters. (**e**) Content of the annotations directory, including quizzes, syntactic annotations and GPT2 word embeddings. (**f**) Content of the annotations directory, including word embeddings and character embeddings of Word2Vec and BERT.

The data collection using the BIDS data representation is available on the OpenNeuro platform at https://openneuro.org/datasets/ds004078 database^11^. All facial features have been removed from the structural images. As shown in Fig. 5a, our data include data description files, the raw fMRI and MEG data collected for each participant in the “sub-*“ folders, the stimuli audio in the “stimuli” folder, the code for the preprocessing and the technical validation of fMRI and MEG in the “code” folder, the preprocessed fMRI and MEG data and the annotations of the stimuli in the “derivatives” folder. More details about these folders are provided below.

### Participant folder

Each participant folder contains four subfolders (Fig. 5b), named “anat”, “dwi”, “func”, and “meg”. The “anat” folder contains the T1 and T2 MRI data. The “dwi” folder contains diffusion MRI data. The “func” folder contains functional MRI data, including 60 runs for the language understanding task and 4 runs for resting state measurements. The “meg” folder contains the MEG data for the 60 language understanding task runs. The json files contain information about the acquisition parameters.

### Stimuli folder

The story audio are provided in the “stimuli” folder (Fig. 5c). The story IDs are consistent with the run IDs in Fig. 5b.

### Derivatives folder

The “derivatives” folder contains the preprocessed fMRI and MEG data for each participant in the “preprocessed_data” subfolder (Fig. 5c), as well as the annotations of stimuli in the “annotations” folder. The preprocessed fMRI data include the 60 language understanding task runs and 4 resting state runs in both the CIFTI and MNI spaces. The preprocessed MEG data contain the 60 language understanding task runs at the sensor level. The annotations of stimuli include the scripts of stories and the quiz questions used in the fMRI and MEG data collection. In addition to this basic information, we provide rich annotations about the language stimuli, including the onsets and offsets of characters and words (Fig. 5d, “time_align” folder), the frequencies of characters and words (Fig. 5d), syntactic annotations (Fig. 5e), and static and dynamic word embeddings (Fig. 5e,f).

### Code folder

The “code” folder contains the code for MRI and MEG preprocessing and technical validation.

## Technical Validation

To verify the quality of the two types of neuroimaging data, first, the accuracy of the quiz responses after each story was calculated to ensure adequate comprehension by the participants. Then, for the MRI data, we checked the head movement for each individual participant and calculated the temporal signal-to-noise ratio (tSNR) and intersubject correlation (ISC). To ensure that the fMRI data encoded language understanding information, we also computed the voxel’s predictability with respect to language information using the neural encoding method. For the MEG data, we used two quality assessment indicators (i.e., ISC and neural entrainment to speech) to evaluate the data quality.

### Behavior results

After the scanning during each story, participants were asked to choose the answers to four-choice comprehension questions (120 questions for fMRI and 120 different questions for MEG) according to the audio they just heard. Participants performed well, with mean accuracy values of 91.18% (SD = 4.21%) and 91.81% (SD = 3.21%) for the fMRI and MEG experiments, respectively (more details are shown in Table 1). An example of a comprehension question is provided below:

“鲸落”是指什么? (what does “Whale fall” mean?)

1. 鲸鱼的种群聚落 (Population settlements of whales)
2. 鲸鱼死后缓缓沉入海底不断被分解消耗 (After death, the whale slowly sank to the bottom of the sea and was continuously decomposed and consumed)
3. 鲸鱼呼吸的水汽四散渐落 (The water vapor from the whale’s breath is scattered and gradually falls)
4. 鲸鱼从海面跃起跌落 (Whales jumped and fell in the sea)

Correct answer: 2

### Analysis of motion

Framewise displacement (FD) measures the frame-to-frame head movement of participants during fMRI scans. The HCP preprocessing pipeline generates three translation parameters and three rotation parameters that represent instantaneous head motion in six directions. Based on these six motion parameters, we calculated the FD of each run by summing the absolute displacements in all six directions between adjacent frames^15^.

**Fig. 6:**
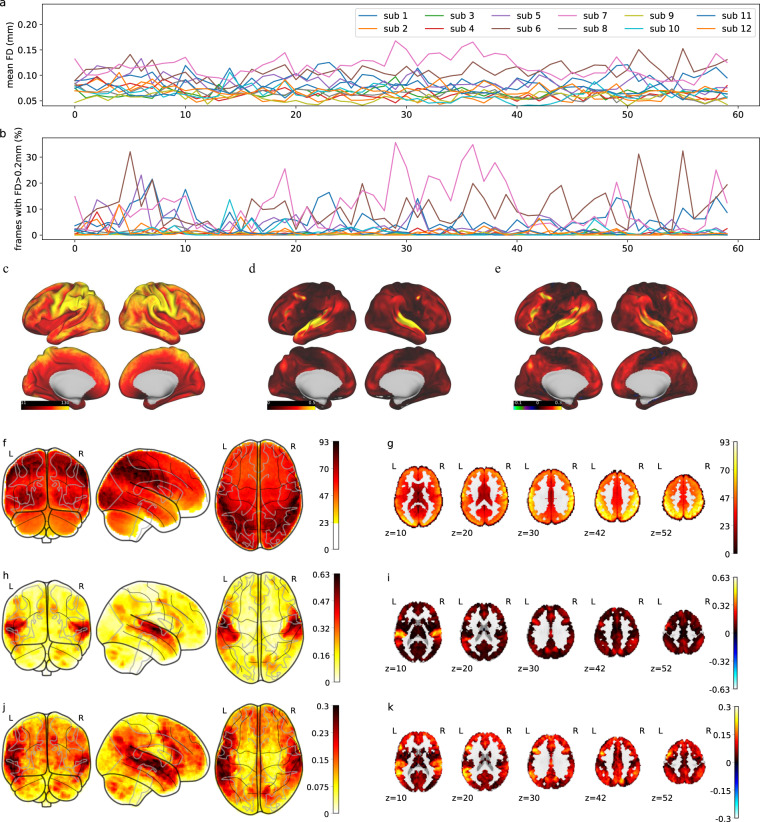
Results of fMRI technical validation. (**a**) Group-level mean FD values for each run and each participant. (**b**) Percentage of each run in which FD > 0.2 mm. (**c**) Group-level mean tSNR in the CIFTI space. (**d**) Group-level mean ISC in the CIFTI space. (**e**) Group-level mean encoding results in the CIFTI space. (**f**) Group-level mean tSNR in the MNI space (glass brain). (**g**) Group-level mean tSNR in the MNI space (axial direction). (**h**) Group-level mean ISC in the MNI space (glass brain). (**i**) Group-level mean ISC in the MNI space (axial direction). (**j**) Group-level mean encoding results in the MNI space (glass brain). (**k**) Group-level mean encoding results in the MNI space (axial direction).

As shown in Fig. 6a, the mean FD values of all participants and all runs are less than 0.2 mm, which indicates that most of the participants had minimal head motion during all runs. Because FD values greater than 0.2 mm are conventionally considered high motion^15^, we also computed the proportion of frames with FD > 0.2 mm in each run. As shown in Fig. 6b, only participants 06 and 07 had more than 20% of frames with FD values greater than 0.2 mm in a few runs. The average percentage of frames in which FD > 0.2 mm within one run across all participants is 3.11%. These results are comparable to or better than those of existing fMRI datasets^16^.

### tSNR

To measure the signal strength at the voxel level, we computed the tSNR for each voxel as the mean of the signal intensity over time divided by its standard deviation. The tSNR was computed for preprocessed functional runs in both the MNI space and CIFTI space. For the MNI space, we applied a gray matter mask with most white matter and ventricle voxels removed. After calculating the tSNR for each individual participant, we generated group tSNR maps by averaging the tSNR values across all the participants. The results are shown in Fig. 6c,f,g. As shown, most brain regions have high tSNR values.

### ISC for fMRI

ISC analysis is often used to evaluate the consistency of the brain response to stimuli across multiple participants. A high ISC indicates the good quality of the brain data. Specifically, for each voxel in each functional run, we computed the correlation between the time series of one participant and the average time series of all the remaining participants. Similar to computing the tSNR, the preprocessed data in both the MNI space and the CIFTI space were computed. The group-level mean ISC is shown in Fig. 6d,h,i. The brain regions with high ISC values are located in the temporal lobe and the frontal lobe, which are typically associated with language processing.

### Neural encoding

Neural encoding calculates the correlation between brain activation and word embeddings to verify whether and where semantic information is encoded^17–19^. To perform neural encoding, we first computed the stimulus representations for each fMRI image using the word embeddings generated by deep language models. According to previous work^20–22^, the middle layers of deep language models better encode multiple language information. Therefore, we chose BERT layer 7 for the word embeddings, convolved the data with a canonical HRF function, and then down-sampled the data to the sampling rate of fMRI. Then, we trained voxel-wise ridge regression models for each participant to predict brain activity based on the stimulus representations. The Pearson correlation between the predicted and actual fMRI signals was computed to evaluate the encoding performance.

Figure 6e,j,k show the encoding results averaged across all participants. The encoding results in language-related brain regions, including the temporal lobe, the parietal lobe, and the frontal lobe, are substantially greater than those of other brain regions, indicating the high quality of our fMRI data.

### ISC for MEG

**Fig. 7:**
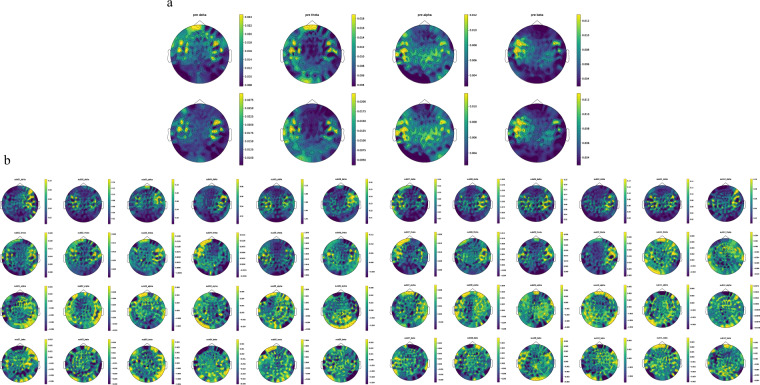
Results of the MEG technical validation. (**a**) ISC results before and after preprocessing. (**b**) Neural entrainment results of four different frequency bands for the 12 participants.

To validate whether our MEG data have a high ISC and whether preprocessing changes the ISC, we calculated the ISC value of the MEG data before and after preprocessing^23^. Specifically, we correlated the amplitude envelopes of bandpass-filtered MEG signals that were correlated between participants in a sensor space in four frequency bands of interest (delta: 1–4 Hz, theta: 4–8 Hz, alpha: 8–13 Hz, beta: 13–30 Hz). First, the MEG signal was filtered for each band. Second, the absolute envelope amplitude of each band was calculated via the Hilbert transform and down-sampled to 1 Hz. Third, for each frequency band, a leave-one participant-out ISC was calculated for the participant left out as the temporal correlation between the envelope amplitude from the participant and the average of the other participants. Finally, the mean ISC was calculated by averaging the ISC across all 12 participants. As shown in Fig. 7a, consistent with previous work, a higher ISC occurred in the delta and theta bands, with a mean and maximum value of (delta: 0.014, 0.431), (theta: 0.009, 0.456), (alpha: 0.007, 0.507), (beta: 0.006, 0.491) respectively over all participants and sensors. Moreover, the ISC in the orbital frontal area, which is related to eye blink or movement, was reduced after preprocessing. In summary, the dataset demonstrated good validity in detecting the ISC, and preprocessing was effective in removing eye movement artifacts.

### Neural entrainment

The phenomenon of the brain activity elicited by listening to speech being time-locked to speech sounds is called neural entrainment, which is an indicator of speech processing. Existing work has found that the less noise there is in the brain data and the more closely the participant pays attention to understanding the speech, the greater the neural entrainment score^24^. Therefore, this score could be used to evaluate the quality of our MEG data. Specifically, we first extracted the temporal envelope from the audio signals and down-sampled them to 50 HZ. Similarly, we down-sampled the preprocessed MEG data to 50 Hz. Then, we used the STRF model^25^ to train an encoder on the speech temporal envelope to predict MEG data filtered into four different frequency bands (delta: 1–4 Hz, theta: 4–8 Hz, alpha: 8–13 Hz, beta: 13–30 Hz) separately. Finally, we estimated the pairwise linear correlation between the predicted and real MEG data. This correlation is often referred to as the predictive power of the STRF model, and it provides a global measure of speech brain tracking. As shown in Fig. 7b, the high predictive power occurred in the lower frequency delta band (with a maximum value of 0.251 and an average value of 0.109 over 12 participants and 306 sensors). Moreover, the sensors with higher predictive power are mainly located in the temporal lobe near the auditory cortices. These findings are consistent with previous studies and verify the quality of our MEG data.

## Data Availability

The scripts for preprocessing the MRI and MEG data as well as those used in the technical validation section are available at https://openneuro.org/datasets/ds004078.
